# Editorial: Emotional intelligence in applied settings: approaches to its theoretical model, measurement, and application

**DOI:** 10.3389/fpsyg.2024.1387152

**Published:** 2024-03-07

**Authors:** Cody Ding, Melissa Ramdas, Marcello Mortillaro

**Affiliations:** ^1^Department of Education Science and Professional Programs, University of Missouri-St. Louis, St. Louis, MO, United States; ^2^Mercy University, Dobbs Ferry, NY, United States; ^3^Swiss Center for Affective Sciences, University of Geneva, Geneva, Switzerland

**Keywords:** emotional intellegence, role of EI in organization and edcuation, role of EI in individuals' lives, academic success, teachers

Peter Salovey and John D. Mayer defined ‘Emotional Intelligence' in 1990 (Salovey and Mayer, 1990), and Daniel Goleman popularized this concept via his book *Emotional Intelligence*, published in 1995 (Goleman, 1995). Since then, many studies have investigated the concept of emotional intelligence and its relationships with behavior and cognition. Although there are debates about the conceptualization and assessment of emotional intelligence (EI) (e.g., Simonet et al., 2021), such as EI as a set of traits or EI as a set of skills, EI proved to be a critical variable worth of consideration for applied research in several domains (e.g., Zeidner et al., 2004; Brackett et al., 2011; Schlegel and Mortillaro, 2019). In this Research Topic, we focused primarily on two specific domains that we think merit particular attention: organizations and education.

The role of EI in organizations has probably been the main focus of attention for the first wave of research following the pioneering publications from Salovey and Mayer (1990). EI has been considered a critical predictor of performance in organizational settings, and several meta-analyses confirmed this assumption (e.g., Joseph and Newman, 2010). Regardless of the conceptualization of EI, authors found that EI has incremental validity over and above the big five and cognitive ability in predicting job performance – at least for jobs high in emotional labor demands (Joseph and Newman, 2010). However, methods and conceptualizations impact the strength of this association (O'Boyle et al., 2011). In two more recent meta-analyses, besides job performance, Miao et al. (2017, 2018) found that EI relates to authentic leadership, job satisfaction, organizational commitment, and, inversely, to turnover intentions, especially when using self-report measures.

The second domain in which EI has been at the center of extensive attention is education, mostly from the perspective of academic success in students. This research, at first, faced several theoretical and methodological issues that raised some criticisms (Humphrey et al., 2007). However, as the field matured, studies found that EI predicted students' success in a range of different education contexts, from secondary education (Sánchez-Álvarez et al., 2020) to performance across all age groups (MacCann et al., 2019), to special professional education contexts like hospitality (Goh and Kim, 2020) or medical school (Libbrecht et al., 2014). Studies focusing on teachers' emotional competencies are relatively less frequent and merit further consideration, but results seem to indicate their critical importance, as we will also read in the present Research Topic.

Our Frontiers Research Topic on *Emotional intelligence in applied settings: approaches to its theoretical model, measurement, and application* recognizes EI as a pivotal area of contemporary psychology. This stance was echoed by the authors in this collection of published articles, stressing the essential positive role of EI in individuals' lives across different contexts. From a general point of view, Manjarres et al. used a bibliometric analysis and literature review of emotional skills and concluded that emotional skills have nowadays become a requirement for success in all scenarios of individuals' actions and a large part of their life cycle.

We present four articles related to the field of education: two contributions that focus on teachers, one in the context of learning a foreign language and one on teachers' life satisfaction, and two on students, one concerning their emotions and the other on academic performance in a specific educational setting. Hu investigated the effects of teacher self-compassion, emotion regulation, and emotional labor strategies on teacher resilience in the English as a foreign language (EFL) context, finding the direct and indirect effects of different EI components on teacher resilience and highlighting EI's role in minimizing difficulties in the challenging field of foreign language instruction. Similarly, Deng et al. examined the effects of three facets of trait EI and two types of effects on rural teachers' general life satisfaction, pointing to the evidence that rural teachers with higher EI are more likely to have positive emotions, enhancing their general life satisfaction. If we move our focus from the teacher to the students, we find the interesting contribution of Xu et al. who used machine learning to identify categories of academic emotion among graduate students based on texts. Finally, Völker et al. focused on the role of EI in predicting academic performance among college students in hospitality education, highlighting how ability EI provides unique advantages beyond cognitive ability and personality traits in predicting academic performance.

Education is strictly related to development, and for this reason, we host two other articles in this collection that are not directly linked to academic performance but investigate the social life and mental health of children and adolescents, themes that can be also considered factors potentially influencing their academic performance. Indeed, Wang et al. studied the effects of emotional awareness on children's social adaptation in terms of emotional development and depression. Zhang et al. investigated the association between expressive flexibility and depressive symptoms among a large sample of adolescents and explored age and gender differences.

We then have two articles related to the organizational context. Robinson et al. hypothesized that a work-contextualized form of EI was positively associated with organizational citizenship behaviors. The pivotal function of EI in job performance is further analyzed by Zhao and Sang, who discussed how trait emotional quotient (EQ) and adversity quotient (AQ) contribute to individuals' objective career success (job position) and subjective career success (organizational commitment) among the general adult population.

In addition to the central theme of the articles mentioned above that emphasizes the applications of EI in social lives across different ages and environments, Audrin and Audrin proposed the concept of “digital emotional intelligence,” going beyond solely EI by integrating digital competence and emotional intelligence in the digital context. This work makes a significant theoretical contribution by highlighting the relevance of digital competence to EI, offering opportunities for future studies.

In this Research Topic, we offer 10 articles that focus on various applications of EI in individuals' social lives, from children to adults. We hope you find something of interest to you in one or more of the 10 articles we present in this collection on the vital role of EI regarding interpersonal relationships and life success. We recognize that other studies may approach these topics differently; likewise, many other EI topics can be and will be discussed in the future. We look forward to continued research on EI to clarify the relationships between different theoretical approaches, suggest ways to integrate them, or identify ideas related to application contexts.

## Author contributions

CD: Writing—original draft. MR: Writing—review & editing. MM: Writing—review & editing.
